# COVID-19 lateral flow test image classification using deep CNN and StyleGAN2

**DOI:** 10.3389/frai.2023.1235204

**Published:** 2024-01-29

**Authors:** Vishnu Pannipulath Venugopal, Lakshmi Babu Saheer, Mahdi Maktabdar Oghaz

**Affiliations:** School of Computing and Information Science, Anglia Ruskin University, Cambridge, United Kingdom

**Keywords:** SARS-CoV-2, lateral flow test, convolutional neural network, StyleGAN2, deep learning, transfer learning

## Abstract

**Introduction:**

Artificial intelligence (AI) in healthcare can enhance clinical workflows and diagnoses, particularly in large-scale operations like COVID-19 mass testing. This study presents a deep Convolutional Neural Network (CNN) model for automated COVID-19 RATD image classification.

**Methods:**

To address the absence of a RATD image dataset, we crowdsourced 900 real-world images focusing on positive and negative cases. Rigorous data augmentation and StyleGAN2-ADA generated simulated images to overcome dataset limitations and class imbalances.

**Results:**

The best CNN model achieved a 93% validation accuracy. Test accuracies were 88% for simulated datasets and 82% for real datasets. Augmenting simulated images during training did not significantly improve real-world test image performance but enhanced simulated test image performance.

**Discussion:**

The findings of this study highlight the potential of the developed model in expediting COVID-19 testing processes and facilitating large-scale testing and tracking systems. The study also underscores the challenges in designing and developing such models, emphasizing the importance of addressing dataset limitations and class imbalances.

**Conclusion:**

This research contributes to the deployment of large-scale testing and tracking systems, offering insights into the potential applications of AI in mitigating outbreaks similar to COVID-19. Future work could focus on refining the model and exploring its adaptability to other healthcare scenarios.

## 1 Introduction

Throughout history, infectious diseases have regularly led to pandemics. In the last four centuries, the deadliest pandemics, including COVID-19, declared a pandemic in March 2020 by the World Health Organization, with a death toll exceeding 6 million people as of 21 May 2022, have profoundly impacted countries worldwide (Roychoudhury et al., 2020; World Health Organisation, 2022). The pandemic has affected populations of all ages, prompting recommendations for measures such as social distancing, face coverings, testing, mass screening, and self-isolation (European Commission, 2020). Various studies such as Burki (2020); Gill and Gray (2020); Lopes-Júnior et al. (2020) emphasize the need for a system to expedite large-scale testing and integrate with contact tracing and healthcare platforms. However, these services necessitate a substantial number of trained personnel, making automation a challenge (Peto et al., 2021; World Health Organization, 2021).

COVID-19 diagnosis primarily involves PCR or rapid antigen/lateral flow tests using nasopharyngeal or oropharyngeal swabs (Wang W. et al., 2020). Reverse transcriptase (RT)-PCR is the gold standard, offering 95% sensitivity and specificity (Cleveland Clinic, 2021), yielding < 5% false positives/negatives (Mayers and Baker, 2020). However, it is time-consuming, costly, and labor-intensive (Wang L. et al., 2020). Rapid antigen tests provide results in 10–20 min and are effective in COVID-19 detection (Dinnes et al., 2021). While interpretation and registration are straightforward on a small scale, they become labor-intensive for health workers when testing millions of patients daily (Ham, 2020).

In this regard, this research introduces an automated RATD validation system using deep convolutional neural network (CNN) for result interpretation in uncontrolled environments (Yamashita et al., 2018). The system simplifies result registration through a web interface and integrates with the national health test and trace system. This automation accelerates result validation, reducing diagnosis and test and trace bottlenecks, enabling more daily tests. It also benefits the public by providing prompt test results, facilitating immediate implementation of preventive measures.

This research's novelty lies in creating a database of diverse RATD images (published as open data) and an automated validation system for faster patient identification. Deep neural network and transfer learning (TL) techniques were employed to classify COVID-19 test images into positive (COVID-19 detected) and negative (COVID-19 not detected) categories. In real-world scenarios, test images vary in quality, resolution, angle, and environmental conditions. A limited availability of real-world COVID-19 test images prompted the use of StyleGAN2-ADA for data augmentation, generating simulated RATD images to enhance the training dataset's size and improve the deep learning model's performance in classifying real-world images.

The rest of the manuscript is organized as follows: Section 2 looks at the existing literature in this domain, Section 3 methodology explains different deep learning models and gives an overview of the experimental setup. The performance of the proposed models is discussed in Section 4, followed by conclusion and future work in Section 5.

## 2 Background

Artificial intelligence (AI) algorithms have been very useful in the field of health and medicine from the diagnosis of cancer tumors to identifying customized treatment protocols for individuals. Attempts have been made to use AI, particularly deep learning techniques to automate the COVID-19 diagnosis and testing workflow and expedite isolation, social distancing, and other mandatory preventive measures (Alazab et al., 2020; Horry et al., 2020; Jamshidi et al., 2020; Pathak et al., 2022; Reshi et al., 2021). Most of this research revolves around the use of CNN's on X-rays and CT images. CNN models exhibit high accuracy and may even surpass human output in most image classification tasks (LeCun et al., 2015; Suzuki, 2017). CNN can contribute toward the detection and prediction of COVID-19 (Alazab et al., 2020), including diagnosis (Jamshidi et al., 2020), based on chest X-ray or CT image classification (Horry et al., 2020; Pathak et al., 2022; Reshi et al., 2021).

Dong et al. (2020) outlined the role of AI in the analysis of medical images including CT, positron emission tomography-CT (PET/CT), lung ultrasound, and magnetic resonance imaging (MRI). The research also highlights the importance and relevancy of AI methods in the analysis of chest X-ray images for the detection of COVID-19. A study by Jamshidi et al. (2020) suggests CNN as a suitable tool for the analysis and classification of medical images and complex non-linear modeling. Since the COVID-19 pandemic, researchers are working on various AI technologies to support healthcare providers in the detection of COVID-19 from X-ray or CT images of patients. Due to the lack of training data, some of these studies used pre-trained models and TL techniques for the detection and classification of COVID-19. A study by Kaheel et al. (2021) used a number of off-the-shelf pre-trained deep models paired with big data for the classification of COVID-19 CT chest images. These models allow accurate identification of suspicious regions in CT images with an accuracy of 95%. This study collects multimodal data from various sources, aiming to enhance access to quality care in low and middle-income countries facing pandemics. The research also investigates different segmentation techniques to improve results. In a similar attempt, Kong and Cheng (2022) propose a COVID-19 chest X-ray image classification model using DenseNet and VGG16 feature fusion in addition to an attention mechanism for deep feature extraction. This study also used ResNet to segment effective image information to achieve accurate classification. The model achieves high average accuracy (98.0% for binary and 97.3% for three-category classification). A study by Zouch et al. (2022) offers a novel method for the automatic detection of COVID-19 using CT and chest X-ray images to assist health systems in diagnosing and managing COVID-19. This study leverages two deep learning models including VGG19 and ResNet50, for early detection of the virus, achieving accuracies of 99.35 and 96.77%, respectively. Similarly, Appari and Kanojia (2022) explore the use of pre-trained deep learning models, including VGG16, for detecting COVID-19 in CT and X-ray images. It emphasizes the appropriate dataset management and investigates methods for the prevention of underfitting and overfitting. They achieved an overall accuracy, precision, and F1-score of 98%. A study by Wang et al. (2022) proposes a new CNN model named MLES-Net, which features a multi-level enhanced sensation module (MLES) for the detection of COVID-19 using X-ray images. This model uses an attention mechanism to focus on key points in information, improving model efficiency. The model utilizes three top-layer structures including a simple FC module, a GAP module, and a GAPFC module—to enhance classification accuracy. The results show the MLES-Net56-GAPFC achieves the highest overall accuracy (95.27%).

Wang L. et al. (2020) developed a COVID-19 diagnosis system based on a deep CNN model, utilizing X-ray images of the patients. The research compared different pre-trained model architectures such as VGG-19 and ResNet-50 to examine the performance and computational efficiency. In a similar study, Reshi et al. (2021) proposed a deep CNN architecture for the detection and classification of COVID-19 based on the chest X-ray images which showed an overall accuracy of 99.5%. This study demonstrated the ability and potential of the CNN models in the classification of COVID-19 chest X-ray images. Similarly, Narin et al. (2021) proposed a model for the detection of COVID-19 using chest X-ray images of infected and healthy individuals. They implemented and assessed three CNN-based models including Inception-v3, ResNet-50, and Inception-ResNet-v2. In total, five-fold cross-validation was used to reduce overfitting. The results of this study show the highest accuracy of 98% which was achieved using the ResNet-50 deep model. A similar study proposed by Sethy and Behera (2020) used the ResNet-50 to extract visual features for detecting COVID-19 infection and then classifying the chest X-ray images with the help of a support vector machine (SVM)-based model. Experiments were performed to compare 13 different DL models for feature extraction. Results show DenseNet201 and ResNet50 achieved the highest accuracy of 93.86 and 95.38%, respectively. A study by Pathak et al. (2022) used transfer learning technique to achieve better results in the classification of COVID-19 CT images. The ResNet-50 network was utilized as the based model to extract the possible features of the CT images, and the deep TL model was trained. This model obtained 96.2 and 93.01% for training and testing accuracy, respectively. A model proposed by Tumuluru et al. (2022) also functioned in detecting the COVID-19 infection from the CT scan images using CNNs. The infection was detected by using the CT scan images with different filters which attained an accuracy of 85.34%, 88.15%, and 87.46%. Several DL models including GAN, extreme learning machine (ELM), and long short-term memory (LSTM) were investigated by Jamshidi et al. (2020), combining the unstructured imaging data with the structured clinical data. These techniques were used to develop a COVID-19 diagnostic system that can analyse mass data in relation to COVID-19 patients. Another study conducted by Shamsi et al. (2021) further looked at the epistemic uncertainty of classification results from different machine learning and statistical models using deep features extracted with different pre-trained models. Ezzat et al. (2020) proposed another similar approach where the GSA-DenseNet121 CNN model paired with gravitational search algorithm (GSA) optimizer to analyse and detect COVID-19 using chest X-ray images. GSA was considered to optimize hyperparameters values of the DenseNet121 architecture for the highest accuracy in diagnosis. The model showed 98% of accuracy in classification. Similarly, Jaiswal et al. (2021) used DenseNet201 for the categorization of the COVID-19 patients based on the chest CT images.

Many other studies including Sahlol et al. (2020), Thuseethan et al. (2021), Alhaj et al. (2022), and Aggarwal et al. (2022) attempted to detect the presence of COVID-19 infection in chest CT and X-ray images. However, the drawbacks of using these imaging techniques are unnecessary exposure to radiation, lack of CT and X-ray machines in resource-poor regions, the need for highly skilled medics for interpretation, and unnecessary travel which increases the risk of transmission and exposure. Lastly, chest CT and X-ray images can only detect the infection at a certain stage when the lung damage has already occurred, making them less effective for early detection. This is where faster diagnosis techniques like rapid antigen testing for disease (RATD) come into play. RATD can expedite COVID-19 detection, mitigating drawbacks of imaging techniques like radiation exposure, resource scarcity, interpretation complexity, and disease transmission risks. Moreover, RATD's ability to detect early-stage infections provides an advantage over late-detection methods like CTs and X-rays.

A study by Wong et al. (2022) presents an automated lateral flow analysis (ALFA) model using CNN and computer vision to analyse images from home-administered COVID-19 lateral flow immunoassays (LFIAs). The system claimed to be more accurate and consistent compared to human interpretation, especially for weak positive results. Such automated reading can enhance the accuracy of mass testing and antibody prevalence studies, reducing false results, and supporting better community surveillance. Similarly, Beggs et al. (2022) present a deep learning approach with a convolutional and multi-scale network to improve the interpretation of rapid antigen tests for COVID-19 delivered via lateral flow devices (LFDs). The algorithm classifies tests into three categories: positive, negative, and void. The model trained on hybridized LFDs and associated PCR results and demonstrates superior performance compared to human interpretation. A study by Vashistha (2022) used computer vision and deep learning models to automate the interpretation of SARS-CoV-2 tests conducted via LFDs. The research involves processing images of the LFD test results to extract a region of interest. Various classification models such as Mask R-CNN were trained on this data to automatically categorize the results as positive, negative, or inconclusive. Similar to the previous study, this method reduces the need for human interpretation, mitigating perception bias. In a similar attempt, Soltan et al. (2021) offered two AI-driven screening tests for COVID-19 including the CURIAL-Lab and CURIAL-Rapide. They utilize clinical data available at patient admission to reduce turnaround times for COVID-19 results and mitigate issues caused by patient status uncertainty. Both models demonstrated consistent performance with CURIAL-Rapide offering results 26.3% faster than LFDs. The combined clinical approach improved the test sensitivity up to 88.2% compared to 56.9% with LFDs only method. The proposed model claimed to potentially decrease the number of negative patients assigned to “COVID-19-suspected” areas. A study by Arumugam et al. (2021) proposed a software approach that utilizes few-shot learning for accurate interpretation of LFAs used in diagnosing diseases. This method only requires a small number of validated images for training, unlike the majority of the deep learning-based methods. This method includes three components: image extraction, a self-supervised encoder, and a few-shot adaptation for generalization to new kits. The study demonstrated high accuracy (99%–100%) in interpreting results from five new COVID-19 LFA kits with just 10–20 images per kit.

A study by Mendels et al. (2021) attempted to automate the interpretation of COVID-19 RATD. This study cropped the test images using planar homography and used a CNN model to detect the bands (either positive or negative). In the real-world scenario, the image captured by the users will not be at a desirable angle making the cropping inaccurate which leads to unreliable model accuracy. These gaps are addressed in the suggested model where the COVID-19 result detection is performed with the images of any background and at varied angles. Pre-trained CNN models are used to diagnose and validate the RATDs along with the implementation of StyleGAN2 for creating fake images to improve the generalization ability of the model with examples of more realistic conditions. StyleGAN2 architecture is a state-of-the-art network for creating naturalistic images. This architecture offers quality, as well as a rapid sampling rate (Viazovetskyi et al., 2020). Researchers from Nvidia introduced SytleGAN2 and proposed two models which are automated and suitable for any generator architecture (Karras et al., 2019). This model could generate new datasets of human faces such as FlickrFaces-HQ and FFHQ which were favorably diverse and of good quality. This architecture has the potential for image generation beyond scale-specific modifications to different styles. Altering the specific style subset will only affect the image in particular aspects. Hence, it is clear from the outcomes of the model proposed by Karras et al. (2019) along with the parallel study by Chen et al. (2018) that StyleGAN2 is much superior to the classic GAN architecture. Similar research conducted by Hermosilla et al. (2021) for face image synthesis used StyleGAN2 by integrating a new modified version of the model that functions with ADA has further helped to identify the state-of-the-art models in image synthesis. Our proposed research benefits the health service system in meeting the COVID-19 testing for a large population using state-of-the-art deep learning techniques for RATD image classification. Similar to other studies presented above that use AI for medical imaging, this proposed research will explore multiple CNN models including vanilla and pre-trained models. The novelty in the approach is to be able to automate this highly time-sensitive task alongside generating a realistic RATD image dataset and achieving good classification performance. As mentioned earlier, the state of the art in synthesizing images using a GAN model correlates with StyleGAN2 as well as StyleGAN2-ADA (Karras et al., 2020) by generating high-quality and high-resolution images. This research will perform a quantitative analysis on the RATD images generated with StyleGAN2-ADA which is expected to provide the best results. The detailed methodology is presented in the next section.

Numerous studies, such as Mendels et al. (2021), Peto et al. (2021), and Shiaelis et al. (2022), have attempted to expedite and enhance the accuracy of interpreting COVID-19 LFA. Although these studies have achieved a degree of success, they all share common limitations, including resolution constraints, the necessity for extensive training data, and issues with accessibility and usability. This suggests that there is still ample room for improvement.

## 3 Methodology

Mass antigen testing is considered to be the primary preventive measure to suppress the spread of the COVID-19 infection (Peto et al., 2021). Tests were/are still advised to be taken regularly by the NHS personnel, school staff and students, key and front-line workers, and others who are flagged by the Test and Trace system (Government UK, 2021). The shortage of trained staff and practitioners at test centers was the major challenge and bottleneck in meeting the demand for mass testing, validating the test results, updating the Test and Trace system, and informing individuals to take the required precautions. To address this problem, this study proposes an automated RATD validation system capable of automatic interpretation of test results using deep CNN models, monitoring the test results using a web application, and integrating these results with the Test and Trace system.

The proposed system utilizes deep CNN models to classify RATD images as either negative or positive cases. Experiments are performed with various CNN models using different hyperparameters in order to identify the best model for RATD classification. Apart from custom vanilla CNN models, the performance of commonly used pre-trained deep CNN models such as NASNetMobile, DenseNet121, and ResNet50 has also been investigated. Following the CNN-based RATD classifier, this study also offers a web application interface that allows operators use the CNN model to validate the RATD results via user interface. This validates the ability of the model to be deployed online and to be integrated into the healthcare provider system workflow. A web API will also be available for the same purpose.

### 3.1 RATD image data set

It can be challenging to build deep learning models if there are no appropriate datasets with public accessibility. Especially if these images are concerned with data protection or privacy or piracy and copyright protection. The datasets required for this research were not available from any online sources or health services as they were concerned with the data privacy of the patients. In such a scenario, the easiest resolution was to manually build a dataset that does not contradict any of the privacy concerns of the public or the test providers.

**Figure 1:**
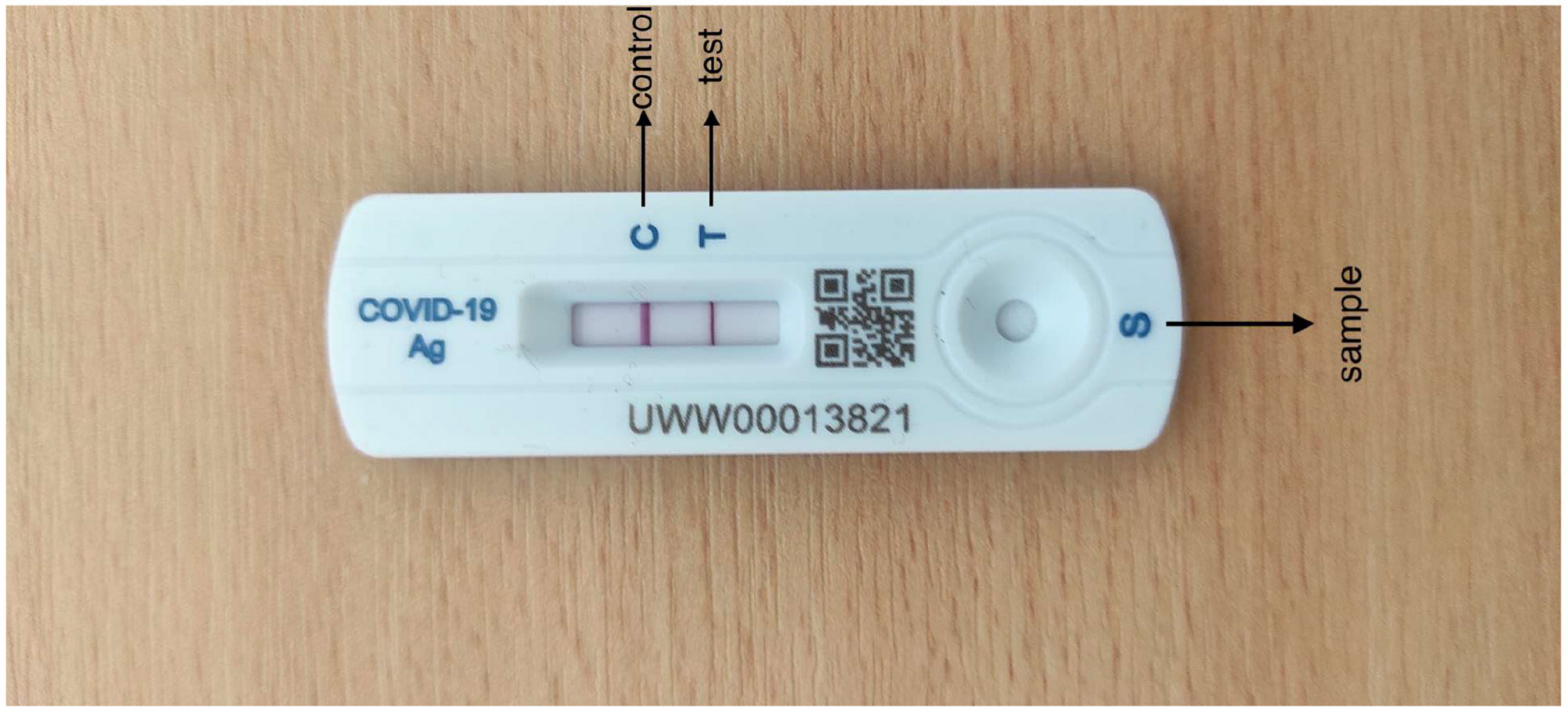
Healgen rapid antigen test device.

Healgen COVID-19 IgG/IgM (Immunoglobulin G/Immunoglobulin M) RATD was utilized which has an accuracy of 88.9% in detecting the SARS-CoV-2 antibody (Corman et al., 2021). Healgen test cassettes are authorized by FDA under EUA for emergency use by individuals or certified laboratories. Their mechanism is to detect both anti-SARS-CoV-2 IgG and IgM antibodies with qualitative or differential detection using the scientific feature of RATD. Antibodies can be diagnosed in 1 to 3 weeks after being infected, as declared by the manufacturer. Healgen RATDs are being used as they were convenient to handle and had the desired performance. In addition, Healgen was the reasonably accessible device on the basis of periodic COVID-19 diagnostic tests. The diagrammatic representation of Healgen RATD is given in Figure 1.

Tests with the RATD were performed referring to the brochure provided by the manufacturer at room temperature following a high standard of stringent biosecurity measures and adequate microbiological techniques. Images of all the tested cassettes were captured on an Android mobile device (Redmi Note 9 Pro and Redmi Note 9 Pro Max) in different backgrounds and in daylight. They were captured at a distance of 20–25 cm away from the reference plane such that the device along with the bands and the background patterns are included in the image with good resolution. Each image has a different resolution according to the angles to enhance the performance of the model which ranges approximately from 1,024 × 768 pixels (3 MB) to 1,600 × 1,200 pixels (5 MB). Images with different resolutions could also aid the training to improve the learning weights as well as the generalization of the deep learning model. The process of testing and image collection lasted nearly 2 months. There were no personally identifying factors on the test device and no conflicts marked by the Organization from where the test kits were collected.

**Figure 2:**
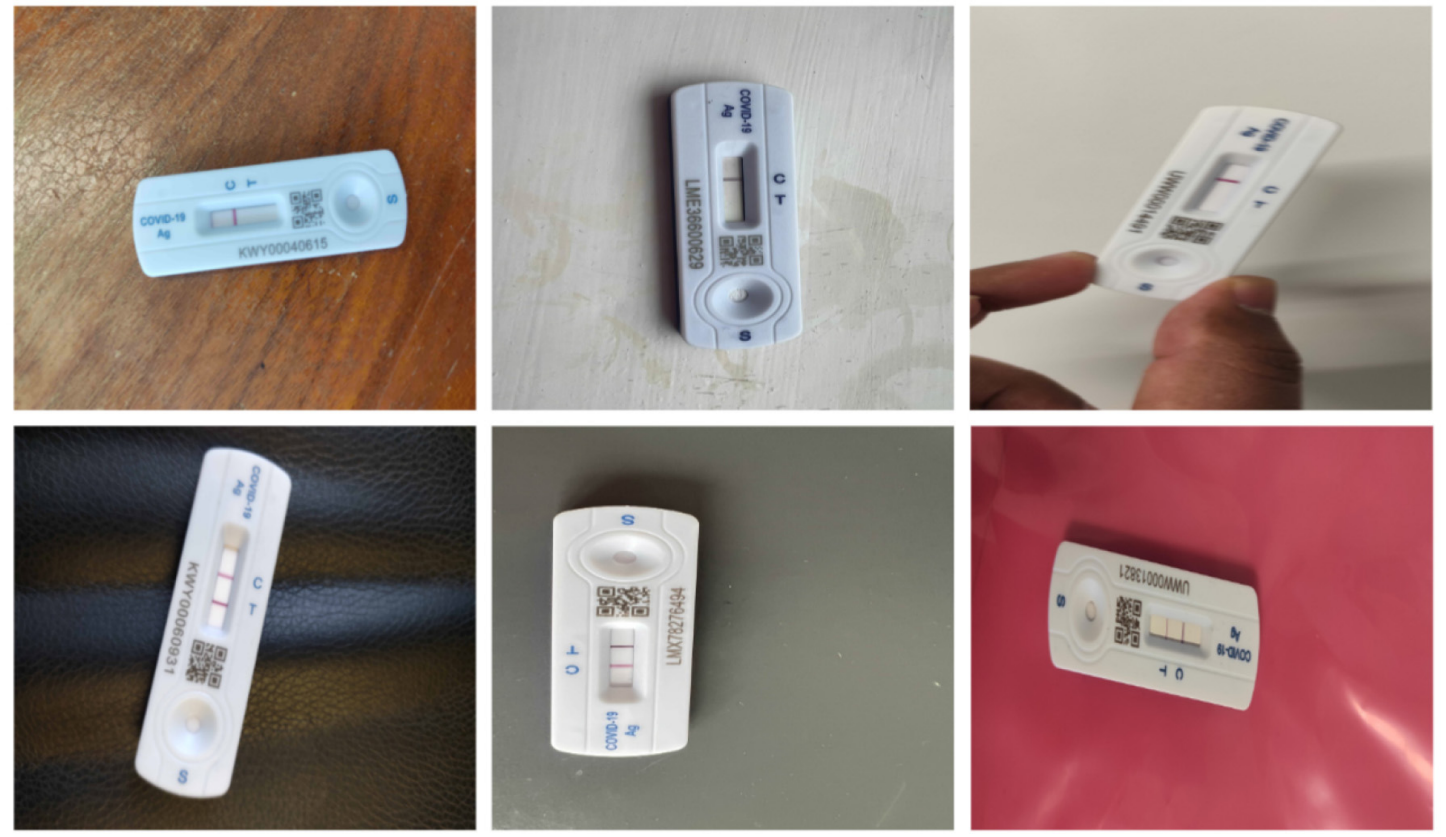
Examples of negative image **(up)** and positive image samples **(down)**.

A total of 900 images were captured for two classes, Positive (COVID-19 detected/True/1) and Negative (COVID-19 not detected/False/0). After the antigen tests, the results were finalized by human reading and labeled as either positive or negative classes. Six-hundred images belong to the Negative class and 300 belong to the Positive class. The images had RATD kits with different backgrounds. In a real-life scenario, once an individual had performed the rapid antigen tests, they can take the picture of the device using a smartphone or any other electronic device. This is followed by uploading the picture to the proposed web application to validate the test results without any human interaction. These images which are uploaded can have different background patterns. For building an efficient system, the train and test images were captured using different backgrounds. Figure 2 shows samples used for training. The StyleGAN2-ADA network was used to generate a fake image dataset for rigorously training and testing the models (explained in Section 3.4.1). The proposed dataset has been made available to the public and research community here. Table 1 shows the breakdown of datasets created.

**Table 1:** Breakdown of data samples for the two classes.

**Dataset**	**Training images (real)**	**Test images (real)**	**Training images (fake)**	**Test images (fake)**
Negative	550	50	100	50
Positive	250	50	100	50

### 3.2 Data preparation

Several data preprocessing approaches such as outlier detection, data scaling, and data transformation are utilized for this research. Experiments were performed involving different dimensions of the images to analyse the most accurate model. The three different dimensions used in these experiments include 224 × 224 × 3, 200 × 200 × 3, and 128 × 128 × 3 for the first, second, and third experiments, respectively. All the images have undergone a two-step verification process to confirm the class to which it is added. Figure 2 show samples from the two classes after labeling the dataset. The images were scaled and normalized before training the models. The image pixel values are normalized between 0 and 1, and the image sizes scaled to different standard sizes as mentioned above.

### 3.3 Experiments

This section summarizes the different deep learning models trained with this dataset. This includes Vanilla CNN, pre-trained standard model architectures fine-tuned using transfer learning with parameter optimisation.

#### 3.3.1 Vanilla CNN models

**Figure 3:**
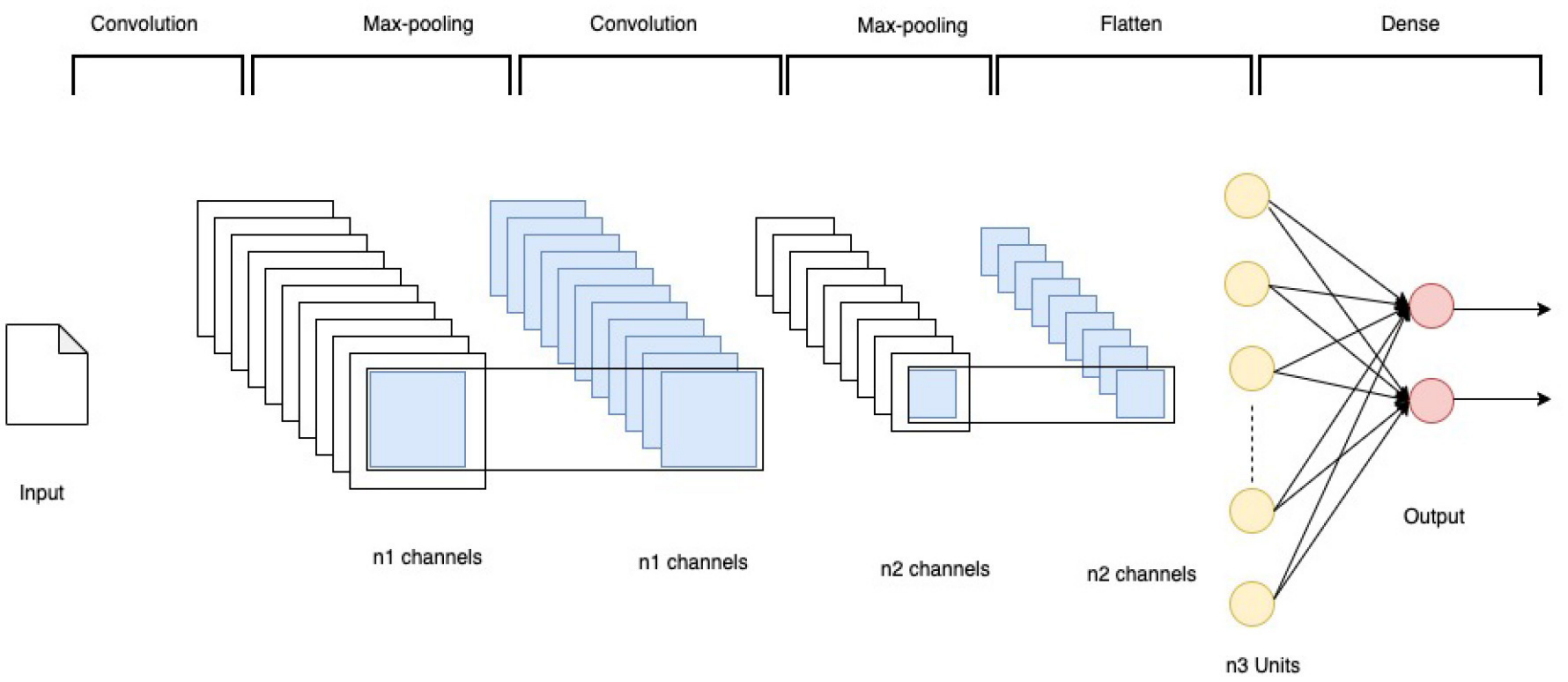
CNN architecture.

This research experimented with several configurations of vanilla CNN models. All parameters were optimized using empirical tests. The final architecture is shown in Figure 3. The models have the network architecture comprising three convolutional layers using filters such as 16, 32, and 64 followed by two dense layers of size 256 and 2. The first block of the convolutional layers utilized a kernel size of 3 × 3. These kernel layers deliver a 2D activation map. Each convolutional layer was followed by a max-pooling layer with a pool size of 2 × 2. All the layers were set with a dropout rate of 0.2. The output from convolution layers is flattened and made compatible with the Dense layers. All the convolutional layers utilized the Rectified Linear Unit RELU as the activation function, and the softmax function was used in the final classification layer.

Experiments were performed using two different optimisation algorithms—Adam and SGD optimisation algorithms. Adam helps to minimize the network parameters, consumes less memory, and is highly efficient in computation (Jais et al., 2019). A range of hyperparameters were optimized for the model as summarized in Table 2.

**Table 2:** Range of hyperparameters tuned for Vanilla CNN.

**Hyperparameter**	**Range/values**
Number of CNN layers	3
CNN filter sizes	16, 32, 64
Dropout_rate	0.2
Learning_rate	0.1–0.0001
Beta_1	0.5–0.9
Beta_2	0.7–0.999
Epsilon	1e-08

The network using Stochastic Gradient Descent as an optimisation algorithm further used a momentum parameter value of 0.9 after initial empirical tests.

#### 3.3.2 Transfer learning

Transfer learning is a machine learning method that uses a pre-trained model mainly as the feature extraction layer. Using the weights of the convolutional layers belonging to the pre-trained model can reduce the number of network parameters to be trained (Kaur and Gandhi, 2020). When this technique is used, only the final dense layers are (re)trained with the input data. In this study, three distinct pre-trained models have been adopted to find the best-performing model in comparison with the default Vanilla CNN models. They are NASNetMobile, DenseNet121, and ResNet50. These models are basically CNNs pre-trained with the ImageNet database that contains more than a million images (Deng et al., 2009). The principal advantages of using transfer learning are resource conservation, along with enhanced efficiency while training the new models with fewer training samples. Each pre-trained network was appended with custom dense layers to redesign the output layer according to the dataset. The dense layer contains a batch normalization technique to reduce the overfitting of the model. Similar to the Vanilla CNN model, ReLU was used as the activation function and Softmax as the output layer activation function. All the pre-trained models used Adam as the optimisation algorithm and same hyperparameters (as shown in the Table 2) for Vanilla CNN is also tuned for these models. Ultimately, the most accurate learning model was chosen depending on the best validation accuracy and loss. The train and validation data split was 80%–20% with a training dataset of 640 images and a validation dataset of 160 images.

### 3.4 Model training

This research experimented with five distinct models: Model A, Model B, Model C, Model D, and Model E. The first two models (Model A and Model B) were implemented using vanilla CNN, and the other three models (Model C, Model D, and Model E) adopted pre-trained CNN models. Experiments were performed with different input image resolutions to find the model with the best accuracy and loss. Various resolutions used were 224 × 224, 200 × 200, and 128 × 128 with RGB channel; however, Model C (NASNetMobile) was only compatible with 224x224 resolution. Hence, there is only a single model trained with this particular resolution. Table 3 demonstrates the training accuracy, training loss, validation accuracy, validation loss, precision, recall, and F1-score for all five models using the training and validation data. Empirical exhaustive parameter and hyperparameter tuning is performed in each of these experiments explained below.

**Table 3:** Experiments and results of training different models.

**Models**	**Test**	**Image size**	**Batch size**	**Params**	**Training Acc. (%)**	**Training loss**	**Val Acc. (%)**	**Val loss**	**Precision**	**Recall**	**F1-score**
Model A	1	224 × 224 × 3	30	12,869,410	88%	0.31	76%	0.46	0.68	0.58	0.63
	2	200 × 200 × 3	30	10,264,354	77%	0.46	72%	0.54	0.76	0.59	0.67
	3	128 × 128 × 3	30	04,218,658	85%	0.34	74%	0.52	0.72	0.52	0.60
Model B	1	224 × 224 × 3	50	12,869,410	70%	0.58	73%	0.55	0.66	0.38	0.48
	2	200 × 200 × 3	50	10,264,354	70%	0.58	70%	0.58	0.79	0.33	0.47
	3	128 × 128 × 3	50	04,218,658	79%	0.45	76%	0.50	0.84	0.82	0.83
Model C	1	224 × 224 × 3	50	04,818,198	89%	0.29	85%	0.38	0.78	0.73	0.75
Model D	1	224 × 224 × 3	50	08,619,074	87%	0.30	**92%**	**0.21**	0.87	**0.87**	**0.87**
	2	200 × 200 × 3	50	08,619,074	**92%**	**0.21**	90%	0.33	0.91	0.80	0.85
	3	128 × 128 × 3	50	08,619,074	82%	0.44	86%	0.41	**0.92**	0.68	0.78
Model E	1	224 × 224 × 3	50	26,221,954	73%	0.60	75%	0.55	0.61	0.62	0.61
	2	200 × 200 × 3	50	26,221,954	76%	0.59	76%	0.52	0.78	0.53	0.63
	3	128 × 128 × 3	50	26,221,954	70%	0.65	72%	0.55	0.68	0.48	0.57

Significant results are in bold.

**Figure 4:**
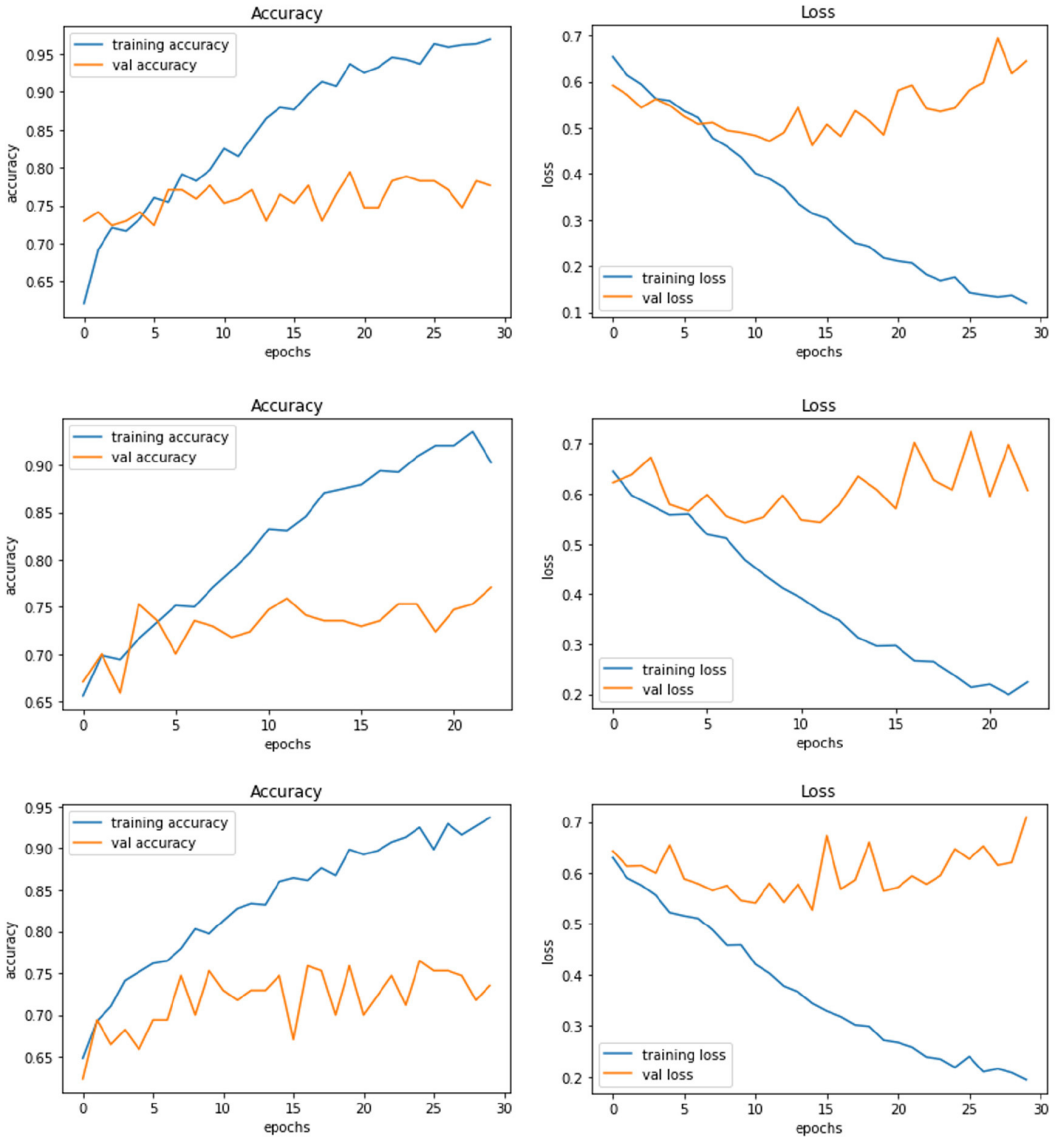
Model A experiment results for 224X224 resolution **(top)**, 200 × 200 resolution **(middle)**, and 128 × 128 resolution **(bottom)**.

Figure 4 demonstrate the three experiments with *Model A* comparing accuracy as well as the loss for training and validation. Among the three experiments, model trained with image resolution 224 × 224 shows better performance with training accuracy of 88% and loss of 0.31, validation accuracy of 76%, and loss of 0.46.

**Figure 5:**
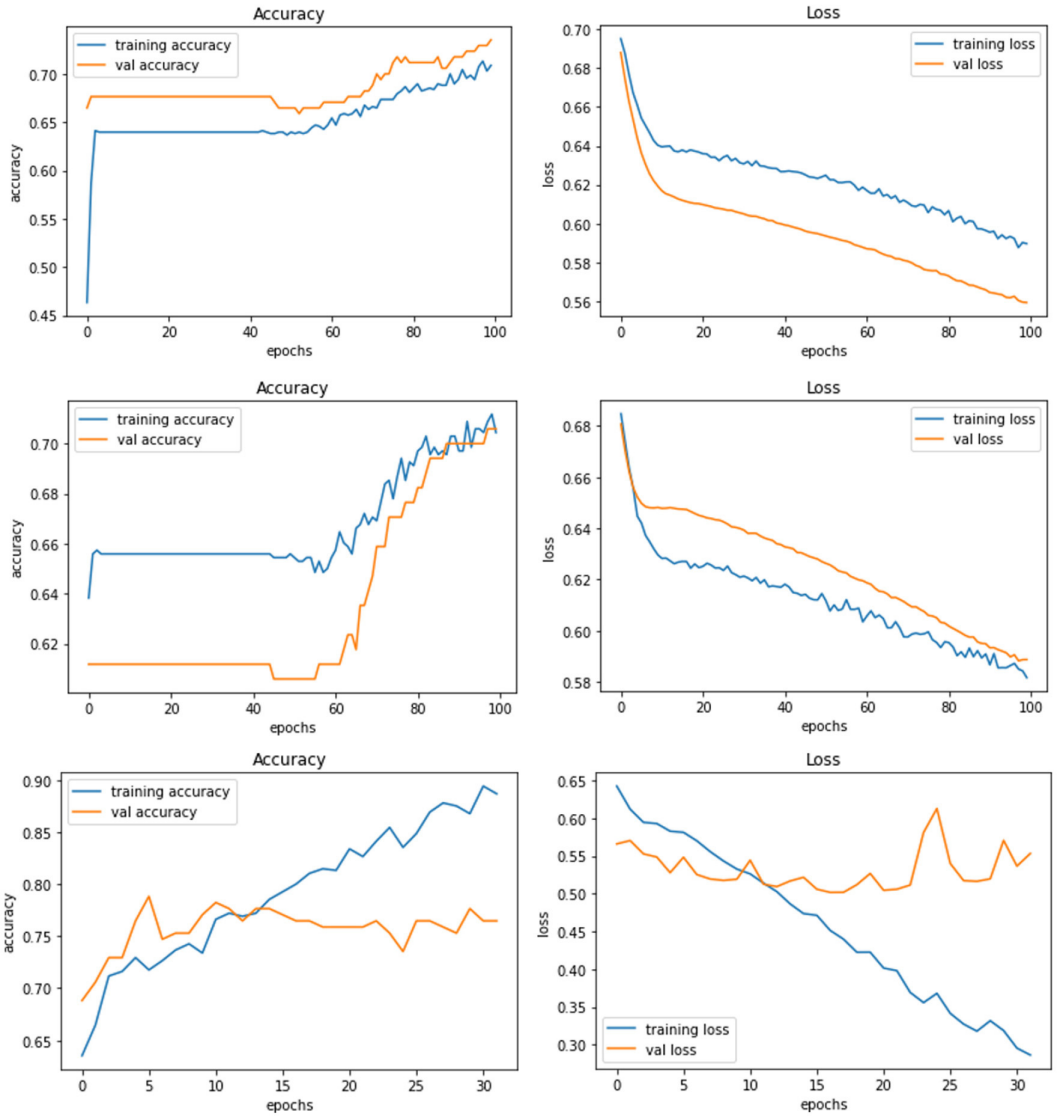
Model B experiment results for 224 × 224 resolution **(top)**, 200 × 200 resolution (middle), and 128 × 128 resolution **(bottom)**.

The network architecture of *Model B* is same as Model A architecture but with SGD as the optimisation algorithm. The model with 224 × 224 RGB image dataset produced the training accuracy of 70% and loss of 0.58, and validation accuracy of 73% with loss of 0.55. Figure 5 demonstrate the loss and accuracy of Model B. The performance difference between different resolutions is not explicitly clear.

**Figure 6:**
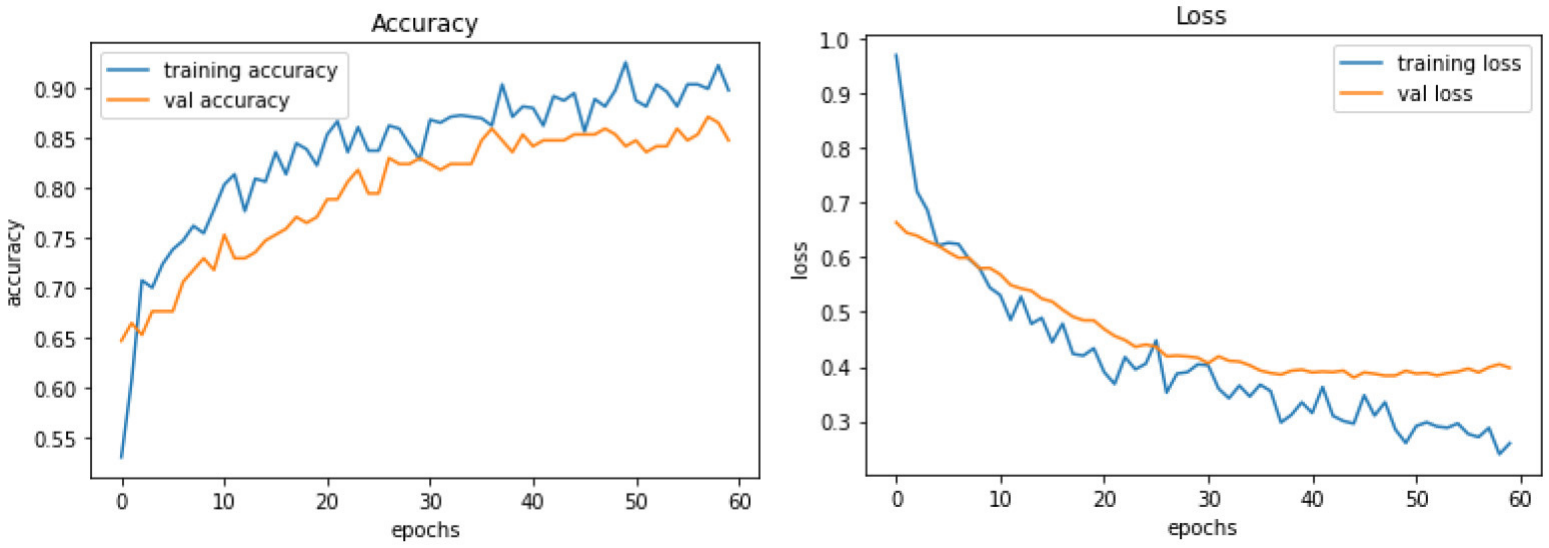
Model C experiment results comparing accuracy and loss of training and validation data for model C.

*Model C* is one of the pre-trained models—NASNetMobile was used to implement the model with a total parameter count of 4,818,198. NASNetMobile is already pre-trained with CIFAR-10 and ImageNet datasets. The model produced training accuracy of 89%, training loss of 0.29, and validation accuracy of 85% with loss of 0.38, which seems to be better compared to performance of Vanilla CNN models (Model A and Model B). Figure 6 shows the loss and accuracy for Model C.

**Figure 7:**
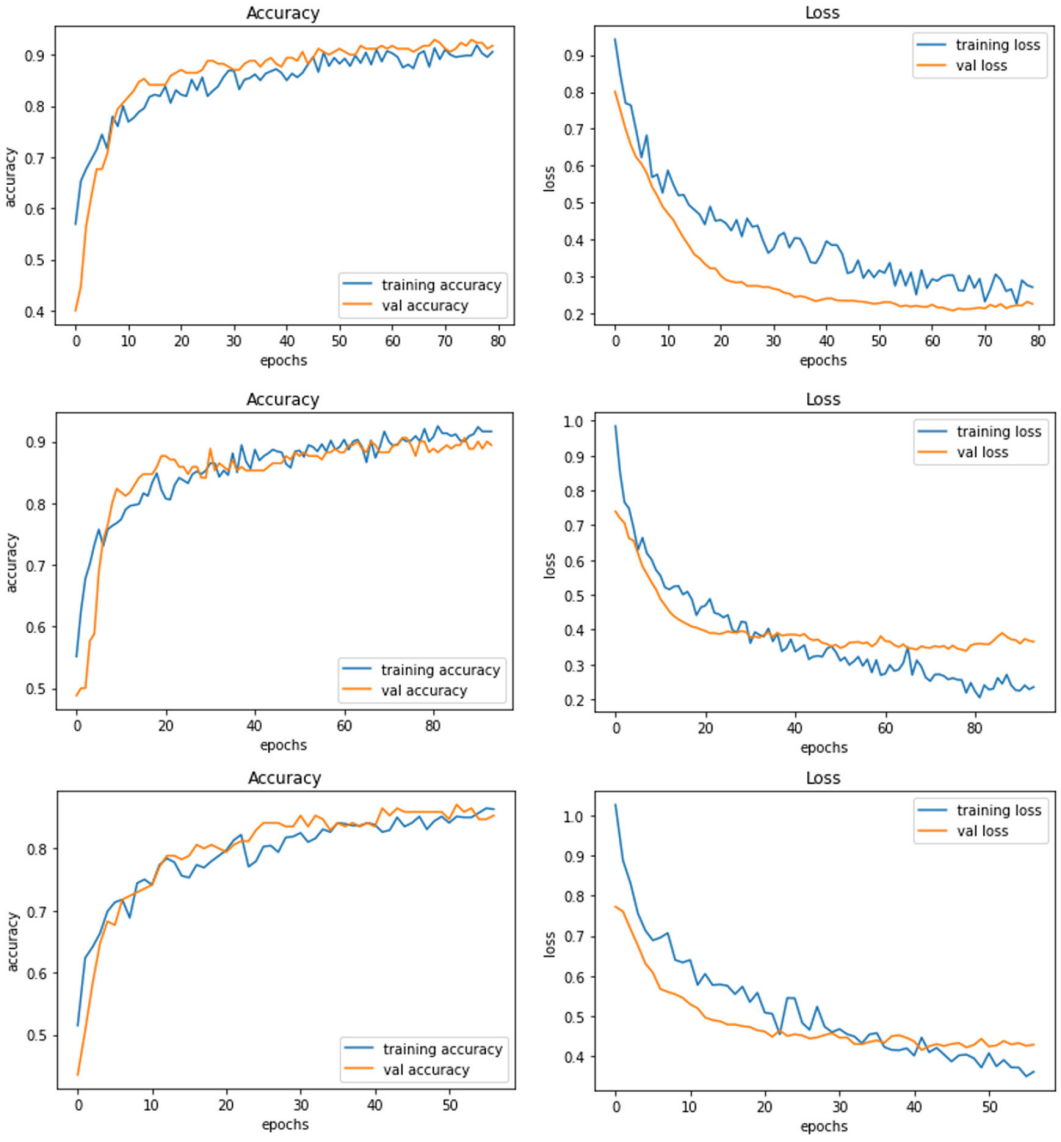
Model D experiment results for 224 × 224 resolution **(top)**, 200 × 200 resolution **(middle)**, and 128 × 128 resolution **(bottom)**.

*Model D* uses another pre-trained model called DenseNet121. This model contains a total parameter count of 8,619,074 which is greater than Model C (4,818,198). Figure 7 demonstrates the three experiments with Model D comparing accuracy as well as the loss of training and validation data. The RGB image size of 224 × 224 generates a training accuracy of 89.85% with loss of 0.29 and higher validation accuracy of 93.53% with a validation loss of 0.20. This model delivers the best performance when compared to all the CNN models and NASNetMobile.

**Figure 8:**
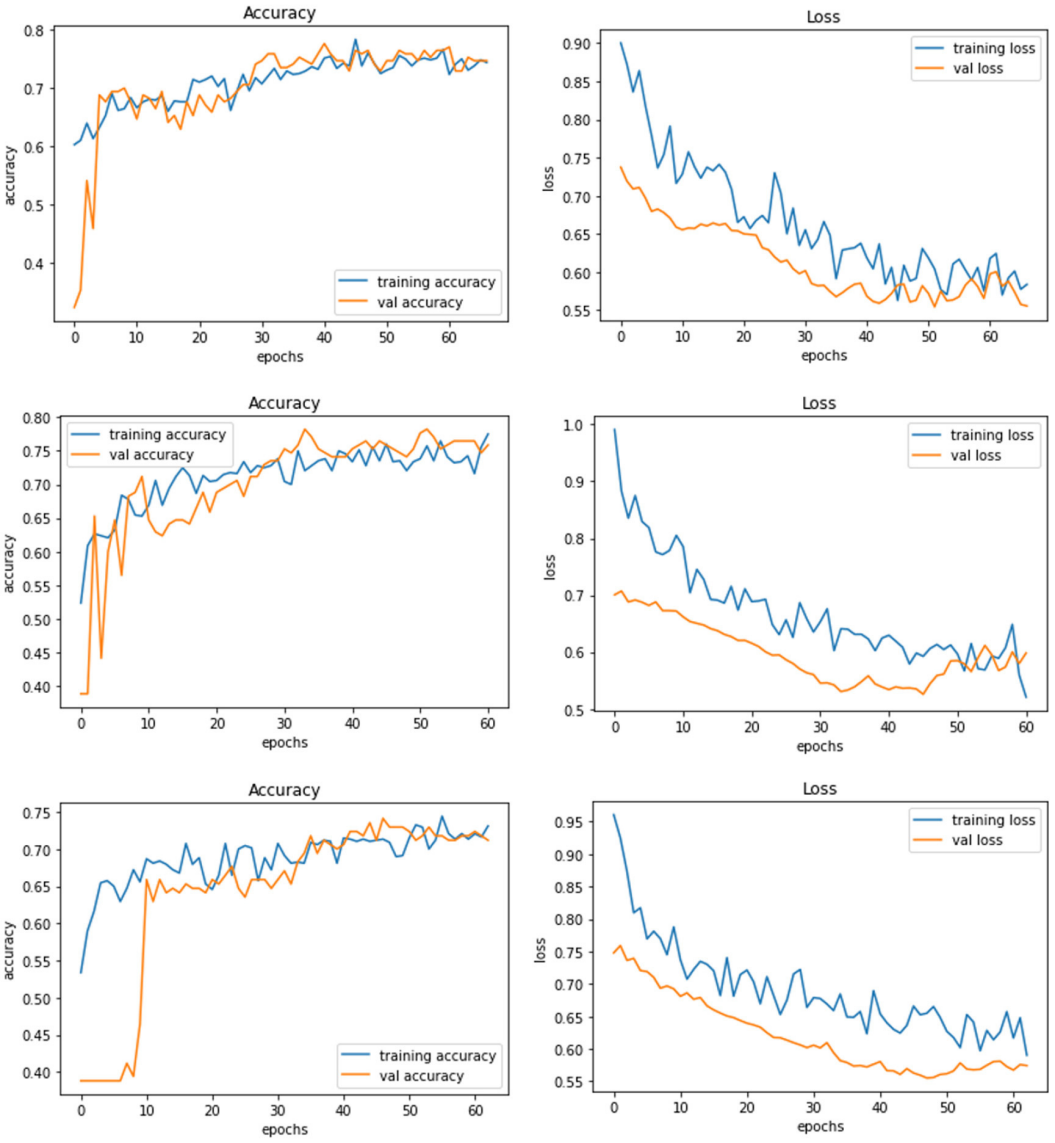
Model E experiment results for 224 × 224 resolution **(top)**, 200 × 200 resolution **(middle)**, and 128 × 128 resolution **(bottom)**.

*Model E* is a transfer learning model with one of the pre-trained models called ResNet50. The total number of parameters of this model was 26,221,954, which is very large compared to other models. Figure 8 demonstrates the three experiments on Model E comparing accuracy as well as the loss for training and validation. When this model is trained with 200 × 200 sized dataset, it produces a training accuracy of 76% with a loss of 0.59 and a validation accuracy of 76% with a loss of 0.52.

As observed from the above experiments, Model D shows the best performance with 224 × 224 image resolution. However, the amount of training dataset was not very large, and perhaps more data could further improve the performance. Hence, data augmentation using StyleGAN2 is performed to generate more images to further train and test the models.

#### 3.4.1 GAN architecture

As mentioned earlier, GAN models could be used to generate images for data augmentation. There are different popular architectures for the GAN topology. A deficient dataset caused the overfitting of the DCGAN where the discriminator learns the training dataset promptly while the dataset is small. StyleGAN2 enhanced with ADA, known as StyleGAN2-ADA can be used to address the problem of overfitting by assessing the discriminator only using magnified images (Karras et al., 2020). Non-invertible data augmentation is transferred to invertible transformations with the help of StyleGAN2-ADA using augmentation probability. Hermosilla et al. (2021) depicted that even though the training dataset is lesser in number, StyleGAN2 functions in generating high-quality and stable images.

**Figure 9:**
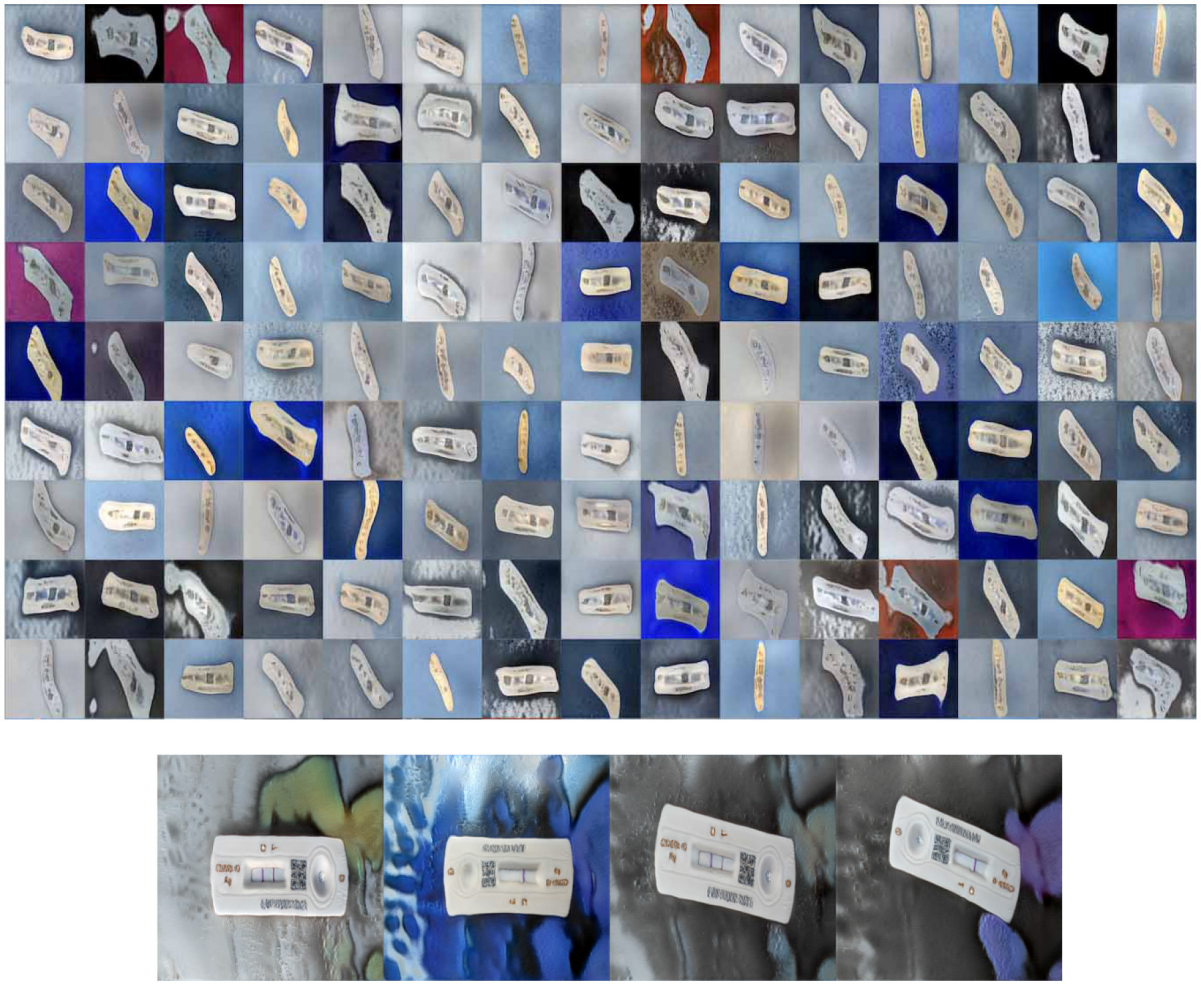
Fake images generated by StyleGAN2-ADA at kimg 161 **(top)** and StyleGAN2-ADA at kimg 640 **(bottom)**.

Pre-trained StyleGAN2 with ADA has been used in these experiments to train and generate images for this research. The training dataset was converted to 512 × 512 RGB images. The suitably transformed images were used to fit the model with the input size parameter of 512 in the training phase. The total number of kimgs (training iterations) required for this training is ~2,500, but due to the limitation of resources and infrastructure, training has been stopped at 640 which itself takes 12.5 h with ~60 Frechet Inception Distance (FID). FID is the metric employed to estimate the grade of the generated images. The snapshot created at the kimg 640 is used to generate the fake images for testing in this research with an image size of 256 × 256. Figure 9 shows the fake images generated at 161 kimg, and kimg-640 configuration. It can be observed that the images look more natural as if taken from different angles and perspectives.

**Figure 10:**
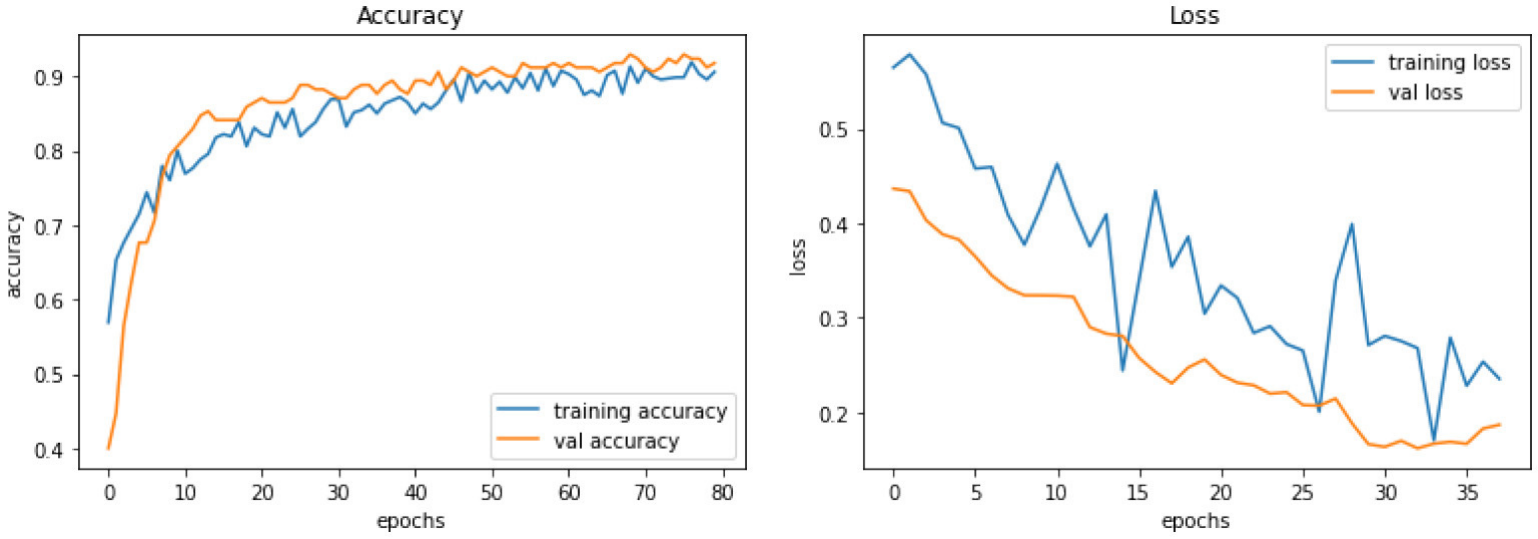
Model DR experiment results comparing accuracy and loss of training and validation data for model DR.

The best-performing model, Model D, was further trained (Model DR) in a transfer learning setup with a fake image dataset generated using StyleGAN2 with a resolution of 224 × 224. The same preprocessing, scaling, and normalization steps as mentioned earlier are followed to retrain the parameters of the model along with empirical hyperparameter optimisation. Figure 9 displays the fake images used. A total of 200 (100 negatives and 100 positives) fake images were used resulting in a more satisfactory performance with a training accuracy of 92% and loss of 0.26, validation accuracy of 93%, and loss of 0.16. Table 4 describes the results of the experiments with Model DR, and Figure 10 demonstrates the accuracy as well as the loss of training and validation datasets for this model. The code associated with the aforementioned deep models is available to the research community here.

**Table 4:** Result of retrained Model D (Model DR).

**Image size**	**224** × **224**
Epochs	33
Training Acc. (%)	92%
Training loss	0.26
Validation Acc. (%)	93%
Validation loss	0.16
Precision	0.92
Recall	0.96
F1-score	0.94

## 4 Results and discussions

The models are further tested using unseen test datasets to estimate the final performance. Three different datasets were used for testing—Real, Fake, and Complex datasets. The real image dataset is similar to the training data, but the image contains different backgrounds and objects alongside the RATD. The fake image dataset was generated using StyleGAN2. Complex image dataset comprise of images that are more challenging with different backgrounds, or with multiple RATD devices placed in various positions in a single image. The training and testing image environments are very different in terms of background colors and objects. The real and fake testing dataset has 100 images each (50 positive and 50 negative class images), whereas the complex dataset has 12 images which add up to 215 images. Six models (Model A, Model B, Model C, Model D, Model DR, and Model E) were tested with real and fake images. Model DR showcased the best performance while training; hence, it was subject to test with a complex image dataset with an input size of 224x224.

### 4.1 Testing with real image dataset

The results of testing the models: Model A, Model B, Model C, Model D, Model E, and Model DR with different image resolutions on the real test images are presented in Table 5. The table presents accuracy, precision, recall, and f1-score for each model. The results indicate that the pre-trained model that was further retrained with simulated data (model DR) shows the best performance across almost all metrics. However, the model is not able to improve mis-detections.

**Table 5:** Testing models with real-image dataset.

	**Exp**	**Image size**	**Accuracy**	**Precision**	**Recall**	**F1-score**	**AUC**	**Sensitivity**	**Specificity**
Model A	1	224 × 224	61 %	0.57	0.86	0.69	0.61	0.36	0.86
	2	200 × 200	59 %	0.56	0.84	0.67	0.58	0.34	0.84
	3	128 × 128	67 %	0.64	0.78	0.70	0.66	0.56	0.78
Model B	1	224 × 224	51 %	0.51	0.86	0.64	0.51	0.16	0.86
	2	200 × 200	52 %	0.51	0.90	0.65	0.52	0.14	0.90
	3	128 × 128	50 %	0.51	0.95	0.67	0.66	0.64	0.68
Model C	1	224 × 224	51 %	0.51	0.86	0.64	0.51	0.16	0.86
Model D	1	224 × 224	79 %	**0.78**	0.80	0.79	0.79	**0.78**	0.80
	2	200 × 200	74 %	0.71	0.80	0.75	0.73	0.68	0.80
	3	128 × 128	71 %	0.67	0.82	0.74	0.72	0.60	0.82
Model E	1	224 × 224	59 %	0.57	0.78	0.66	0.60	0.40	0.78
	2	200 × 200	57 %	0.56	0.70	0.62	0.57	0.44	0.70
	3	128 × 128	57 %	0.55	0.84	0.66	0.58	0.30	0.84
Model DR	1	224 × 224	**82** %	0.74	**0.98**	**0.84**	**0.82**	0.66	**0.98**

Significant results are in bold.

### 4.2 Testing with fake image dataset

The results of testing the models: Model A, Model B, Model C, Model D, Model E, and Model DR, with different image resolutions on the fake test images are presented in Table 6. The table presents accuracy, precision, recall, and f1-score for each model. The confusion matrices for all six models are presented in Figures 1–12 in the Supplementary file. The results indicate that the pre-trained model further retrained with simulated data, Model DR shows the best performance with almost all metrics. Further as seen in the confusion matrix, Figure 6 of the Supplementary file, the model is able to improve performance across both positive and negative classes for fake images. Finally, after testing the six models (Model A, Model B, Model C, Model D, Model E, and Model DR) with real and fake images, Model DR has delivered outstanding performance. Hence, this model was tested again using real and complex RATD images shown in Figure 3 of the Supplementary file. The paired *t*-test result presented in Table 7 shows there is a significant accuracy improvement in model DR when compared to other models tested in this study. *p*-value of 0.05 and degree of freedom of 10 − 1 = 9 (10-fold cross-validation) have been used in these experiments.

**Table 6:** Testing models with fake-image dataset.

	**Exp**	**Image size**	**Accuracy**	**Precision**	**Recall**	**F1-score**	**AUC**	**Sensitivity**	**Specificity**
Model A	1	224 × 224	70 %	0.67	0.78	0.72	0.71	0.62	0.78
	2	200 × 200	73 %	0.71	0.78	0.74	0.74	0.68	0.78
	3	128 × 128	72 %	0.74	0.68	0.71	0.73	0.76	0.68
Model B	1	224 × 224	63 %	0.58	0.96	0.72	0.63	0.30	0.96
	2	200 × 200	61 %	0.56	0.98	0.72	0.61	0.24	**0.98**
	3	128 × 128	50 %	0.50	0.95	0.67	0.62	0.76	0.46
Model C	1	224 × 224	64 %	0.63	0.66	0.65	0.65	0.62	0.66
Model D	1	224 × 224	77 %	0.81	0.70	0.75	0.78	0.84	0.70
	2	200 × 200	76 %	0.80	0.70	0.74	0.76	0.82	0.70
	3	128 × 128	74 %	0.76	0.70	0.73	0.74	0.78	0.70
Model E	1	224 × 224	61 %	0.64	0.50	0.56	0.61	0.72	0.50
	2	200 × 200	66 %	0.62	0.84	0.71	0.67	0.48	0.84
	3	128 × 128	61 %	0.57	0.88	0.69	0.62	0.34	0.88
Model DR	1	224 × 224	**88** %	**0.88**	**0.98**	**0.88**	**0.89**	**0.88**	0.88

Significant results are in bold.

**Table 7:** Paired *t*-test results against DR model using fake-image dataset.

**Paired *t*-test**	**DoF**	***p*-value**	***t*-value**	**Significance**
**Model (A, DR)**	**9**	**0.05**	**14.35**	**Yes**
Model (B, DR)	9	0.05	19.12	Yes
Model (C, DR)	9	0.05	19.07	Yes
Model (D, DR)	9	0.05	11.63	Yes
Model (E, DR)	9	0.05	20.18	Yes

**Figure 11:**
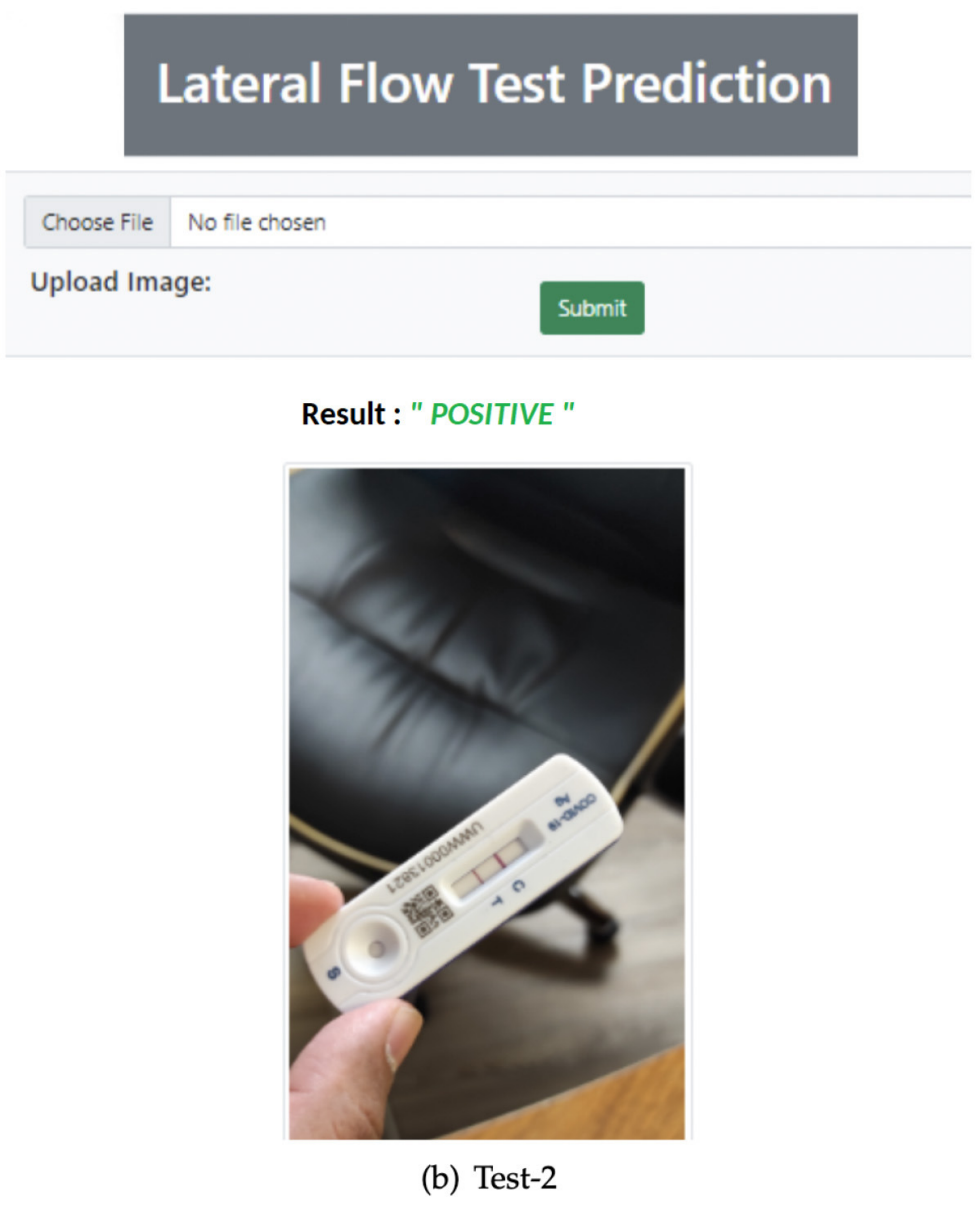
Accurately validated positive RATD image using Web App.

To validate the real-time RATD images, an application has been implemented. Model DR was integrated with this application to validate the input images. A web UI is provided with an API that provides this service to other applications. The application has been implemented using one of python's web frameworks called Flask which helps to foster further development. For the web view, an HTML template is used along with a python script. The complex image dataset was tested using the implemented UI. Figure 11 shows the complete UI of this web application with validation of a positive RATD image.

**Figure 12:**
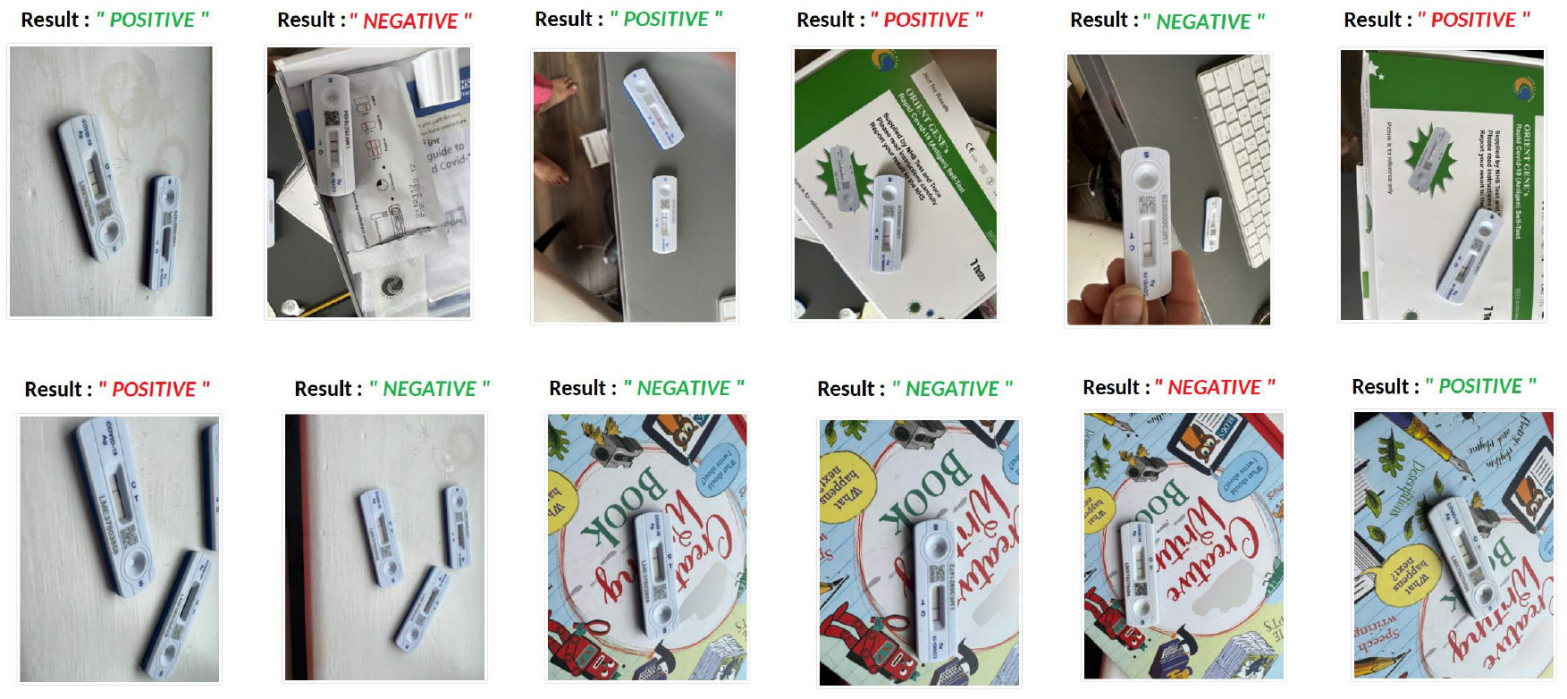
Model DR output with complex images.

The complex image dataset was tested using the implemented UI which showed the results as in Figure 12. The images with headings marked in green color are those with accurate predictions and those with red are inaccurate. The model in general seems to be able to cope with different angles and some of the challenging backgrounds. Some of these images are really challenging like the ones with the RATD package with the device picture in the background depicting a conflicting result. Some images in the dataset had multiple devices; however, training images only consisted of single devices, and still 60% of this challenging dataset was accurately classified. Furthermore, few of these images had both classes (Positive and Negative) in a single image which is challenging even for a human annotator. verall Model DR seems to perform decently and could be further improved in the future.

### 4.3 Comparison

A number of other studies including Arumugam et al. (2021), Beggs et al. (2022), Vashistha (2022) have attempted to utilize AI, machine learning, and computer vision techniques to accelerate the interpretation of RATD images, as well as enhance diagnostic accuracy and sensitivity. Due to the variations in the dataset used and experimental setup, these studies cannot be directly and objectively compared with the proposed work. Nevertheless, the following section offers insight into these studies and describes how they stack up against the proposed work in this study. Arumugam et al. (2021) utilize few-shot learning technique for accurate interpretation and diagnosis of COVID-19 LFAs. Unlike the majority of deep learning-based methods, few-shot learning only requires a small number of validated and training images. The proposed method in this study consists of three components: image extraction, a self-supervised encoder, and a few-shot adaptation for better generalization. The training set in this study consists of 383 images, while the testing set includes 254 images. Additionally, this study used variational autoencoder to generate synthetic images and further evaluate the model performance. The study demonstrated high accuracy of 99.6 in interpreting COVID-19 LFA kits. While this study achieved exceptional results in terms of accuracy, it relies on highly supervised and time-consuming preprocessing operations to extract the LFA membrane region which contrasts with the approach we propose in this study. The proposed approach in our study requires no manual image enhancement or preprocessing which significantly reduces the time and labor costs, streamlines the process, and ultimately allows for a more efficient and scalable solution.

Beggs et al. (2022) also offer a platform named MagnifEye for automated interpretation of COVID-19 LFA results. This study uses a combination of CNN and a multi-scale network to identify and classify the LFA read area into three classes including Positive, Negative, and Void. This study employed t Assisted Testing Sites (ATS) dataset which consists of 59,164 LFA kit images, however, from which only 126 (< 0.2%) samples were COVID-19 positive. Although this research attempted to mitigate the negative impact of the heavily imbalanced dataset using techniques like bootstrapping and confidence intervals, these methods may not be sufficient to fully address the issue, potentially leading to biased results or overfitting. The proposed approach in our study countered the data imbalance issue by generating fake test images using generative models like StyleGAN2-ADA which is proven to be more effective compared to classic statistical methods. Vashistha (2022) is also another closely related study that attempted to use computer vision and deep learning models to automate the interpretation of COVID-19 LFA tests. This study employed a pre-trained (COCO) Mask R-CNN model fine-tuned on a relatively small dataset of 439 LFA test images. Similar to Arumugam et al. (2021), this study relies on heavy annotation, human interpretation, and preprocessing operations to extract the LFA membrane region which contrasts with the fully automated approach we propose in this study.

Unlike the aforementioned studies, our proposed method harnesses the capabilities of potent CNN generative models, specifically StyleGAN2-ADA. This approach serves to augment the pool of training sample images, ultimately enhancing the model's capacity for generalization and improving its overall accuracy. This study makes a significant impact on public healthcare by expediting large-scale trace and testing processes. A typical rapid antigen test typically requires 15–20 min to yield results, a delay that often results in substantial backlogs and lengthy waiting lists, adding to the workload at testing centers. The proposed system, however, has the potential to alleviate this burden significantly. It operates by autonomously analyzing and classifying the test results from the test device images and then promptly relaying this information to patients via a mobile app. Unlike other studies, the proposed system eliminates the need for human intervention. Even in the case of RATD tests, human analysts is required to visually inspect the images of RATD devices to determine whether the results were positive or negative and update the system. We have not only eliminated the necessity for human labor but also expedited the process of identifying patient needs and controlling the spread of disease, thereby reducing associated costs. Furthermore, it has the capacity to streamline the incorporation of contact tracing and tracking, enhancing the overall efficiency and effectiveness of the testing process. By expediting result delivery and bolstering public health efforts through contact tracing, this innovative system promises to play a pivotal role in the battle against contagious diseases. Table 8 provides a brief summary of the comparison between state-of-the-art COVID-19 LFA classification studies and the work proposed in this study.

**Table 8:** Comparison of the state-of-the-art COVID-19 LFA classification studies and the proposed work.

**References**	**Dataset**	**Method**	**Accuracy**	**Cons**
Arumugam et al. (2021)	637	Few-shot learning CNN Variational autoencoder	99.6	Highly supervised and time-consuming preprocessing operations unscalable
Beggs et al. (2022)	59,164	CNN Multi-scale network	98.60	Heavily imbalance High class bias
Vashistha (2022)	439	Pre-trained Mask R-CNN Fine-tuned	92.6	Highly supervised Manual preprocessing
Proposed work	900	CNN StyleGAN2-ADA	88	Could be enhanced for multi-label output to detect multiple devices in an image

## 5 Conclusion and future work

COVID-19 is the pandemic of the 21st century which is still ongoing in most countries with new variants rapidly spreading and iterating through society. Mass testing, as well as vaccination, seems to be the only way to prevent the spread and death rate for this pandemic. Being a highly infectious disease that is still spreading through the entire global population, healthcare workers are still struggling to identify positive cases. The RATD, lateral flow devise seems to be an affordable, rapid-action, and scalable technique to perform the testing of this viral infection. Due to the infectious nature of the disease and the lack of manpower in the healthcare sector, it is important to automate the diagnosis and isolation of patients. This research introduces a system for the automatic validation of RATD images with the help of deep learning and transfer learning techniques. The study was also successful in developing a web application integrating with the best-performing model (Model DR) with a validation accuracy of 93% and test accuracy of 82 and 88% for real and simulated unseen datasets.

This study performed extensive experiments with different categories of deep learning models including Vanilla CNN and several pre-trained CNN models (that can avoid overfitting) using different parameters and different input image resolutions. As expected, the pre-trained models exhibited better performance in classifying the unseen data (Yamashita et al., 2018), and particularly pre-trained model with DenseNet121 could deliver the best performance compared to all other models.

One of the main challenges encountered during this research was the inadequate amount of data for training and testing as the datasets were not publicly available due to the sensitive nature of the data. This motivated the authors to generate a custom dataset using live testing and images taken through mobile devices. However, the availability of the positive class (COVID-19 detected) was another challenge to deal with as there was limited access to patients. Research also looked at simulating images using state-of-the-art GAN models. Initially, DCGAN was used, but it led to noisy images due to overfitting as the training images were fewer in number. Hence, leveraging the StyleGAN2-ADA network helped in creating the fake realistic images for better training and exhaustive testing.

These experiments also discovered the necessity of accurate images for these tasks. Not all images in the dataset were captured with adequate clarity (Blurred images) which affected the prediction of test images leading to incorrect predictions. Model DR, a pre-trained model further retrained with real and simulated images emerged to be the most accurate model when tested with real, fake, and complex test images. The fake image confusion matrix of Model DR (Figure 12 of the Supplementary file) exhibited more true predictions for both classes compared to the original pretrained model, Model D (Figure 10 of the Supplementary file). However while comparing the real image confusion matrices of Model DR (Figure 6 of the Supplementary file) with Model D (Figure 4 of the Supplementary file), the Negative class improved, but the Positive class shows only comparable performance. Hence, it could be concluded that training models with simulated image datasets may not considerably improve the performance for real test images, perhaps only for the simulated test images. Tests were also performed with challenging real-world images with multiple RATD and other distracting pictures in the background. Incorporating an object detection algorithm could help to improve the validation of multiple RATDs in a single image, thus covering these complex scenarios. Furthermore, the research can be further extended by experimenting with other pre-trained models for transfer learning and further optimizing the parameters for better generalization. The proposed model leverages powerful CNN generative models like StyleGAN2-ADA, expanding the training image pool, enhancing generalization, and improving accuracy. This study significantly accelerates large-scale tracing and testing in public healthcare. Rapid antigen tests typically take 15–20 min, causing backlogs and delays at testing centers. Our system autonomously analyses and categorizes test results from device images, relaying information to patients via a mobile app, eliminating the need for human intervention. This not only reduces labor costs but also speeds up disease identification and control. It also streamlines contact tracing, boosting testing efficiency and public health efforts. This innovative system plays a pivotal role in fighting contagious diseases by expediting result delivery and enhancing contact tracing.

This study is subject to several limitations that could be addressed in future research endeavors. During the course of this study, our research was hampered by a relatively limited number of training samples, which had a substantial impact on our findings. To enhance the accuracy of the proposed model and reduce its dependence on artificial intelligence-generated images, it is crucial to include a more extensive dataset in subsequent study. Furthermore, a notable limitation lies in the model's sensitivity to lighting conditions. This sensitivity stems from the primary training of our models on images captured in well-lit conditions, predominantly during daylight. Consequently, the model exhibits a bias toward well-illuminated scenarios, resulting in subpar performance when analyzing images taken in low-light environments. This limitation can be ameliorated by diversifying the training dataset to encompass a broader range of lighting conditions. Additionally, the current model falls short in detecting multiple instances of RATD in a single image. Addressing this limitation should be a priority in future research to make the model more versatile and comprehensive. Moreover, the integration of localization alongside classification is another vital aspect that warrants attention in forthcoming study. This enhancement can enhance the model's ability to pinpoint and classify RATD effectively.

## Data availability statement

The raw data supporting the conclusions of this article will be made available by the authors, without undue reservation.

## Ethics statement

Ethical approval was not required for the study involving human participants in accordance with the local legislation and institutional requirements. The activities described within the article were carried out by the authors themselves, all of whom agreed to participate in the research and publish the findings.

## Author contributions

All authors listed have made a substantial, direct, and intellectual contribution to the work and approved it for publication.
