# Supplementary material for: COVID-19 lateral flow test image classification using deep CNN and StyleGAN2
Source: Front Artif Intell. 2024 Jan 29;6:1235204. doi: 10.3389/frai.2023.1235204 (PMC10860423; doi:10.3389/frai.2023.1235204)
Supplement: Supplementary file 1 [file Image_1.pdf]

Supplementary File 1:

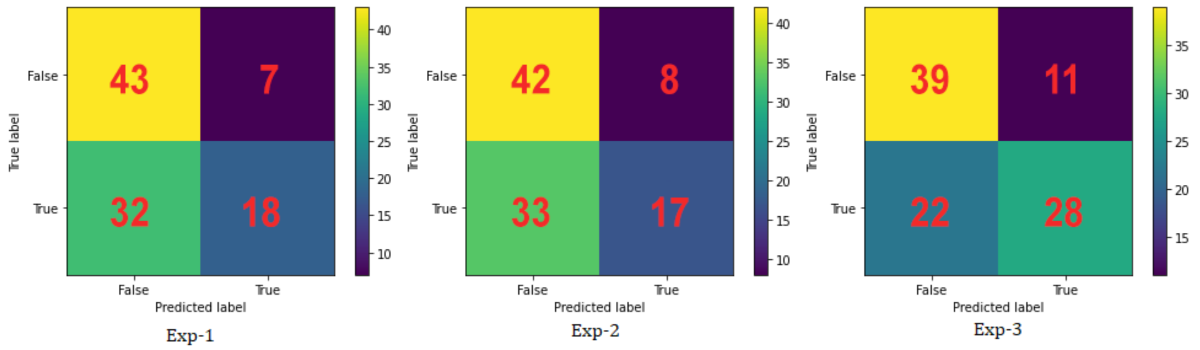

Figure 1. Model A Confusion Matrices using Real Image Dataset

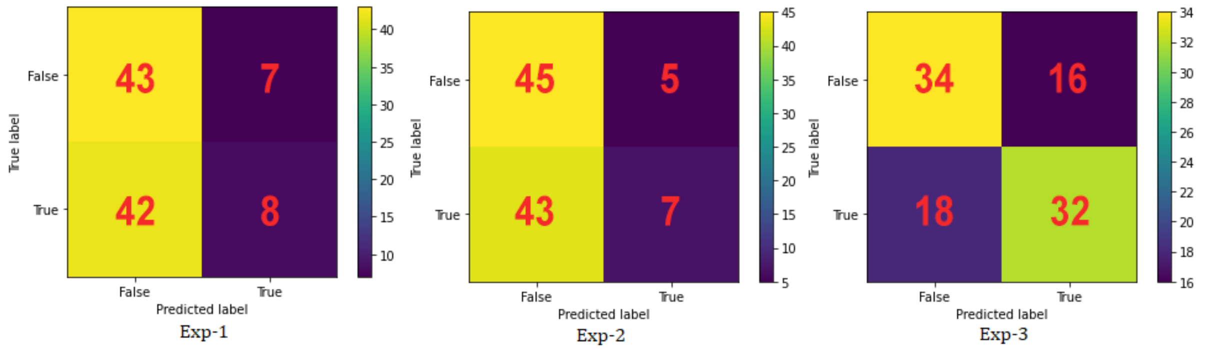

Figure 2. Model B Confusion Matrices using Real Image Dataset

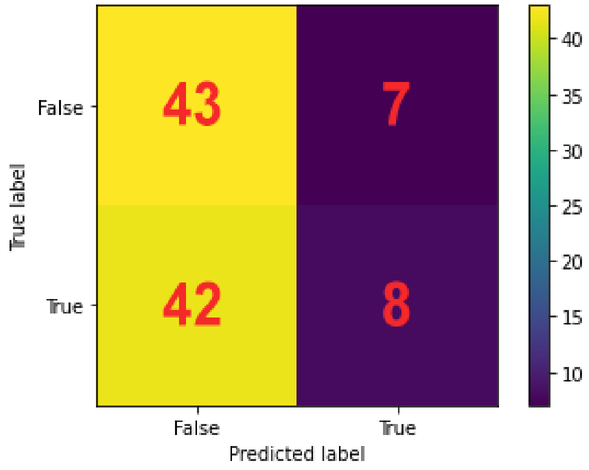

Figure 3. Model C Confusion Matrix using Real Image Dataset

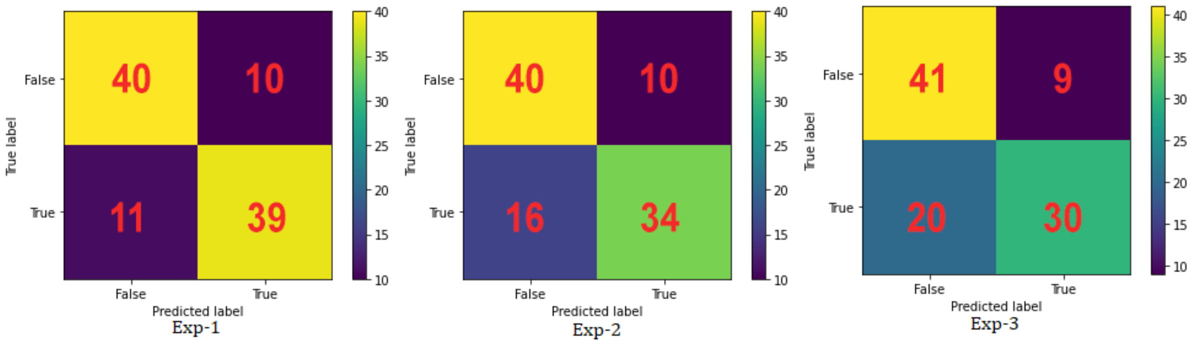

Figure 4. Model D Confusion Matrices using Real Image Dataset

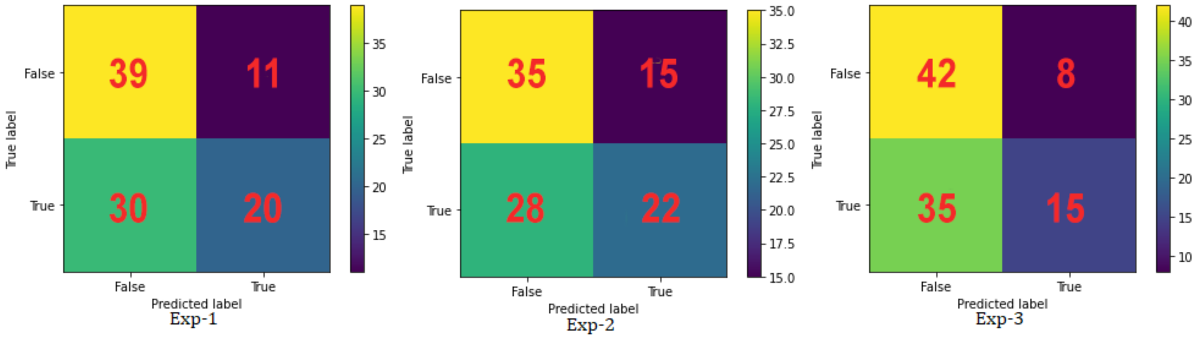

Figure 5. Model E Confusion Matrices using Real Image Dataset

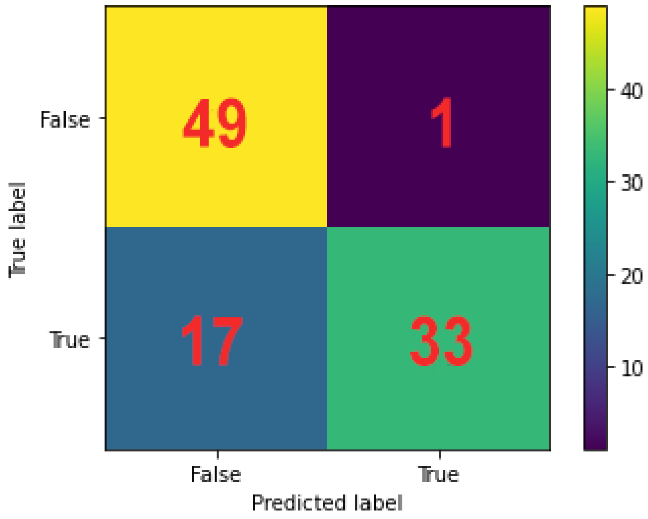

Figure 6. Model DR Confusion Matrix using Real Image Dataset

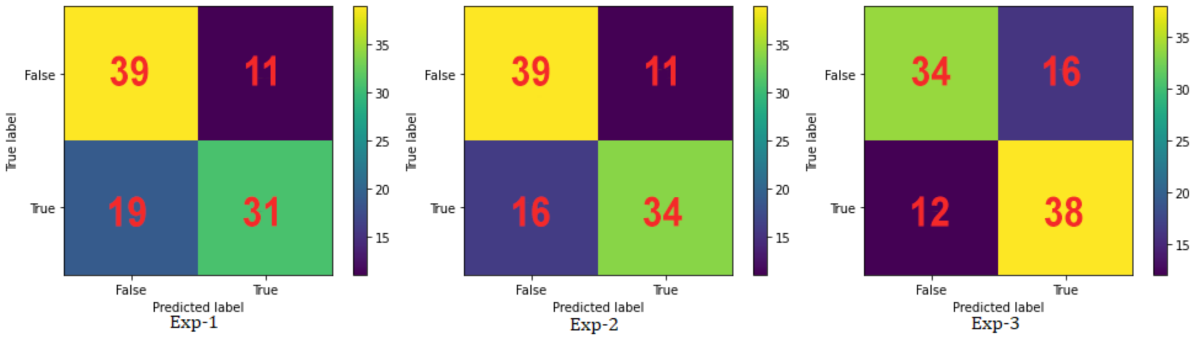

Figure 7. Model A Confusion Matrices using Fake Image Dataset

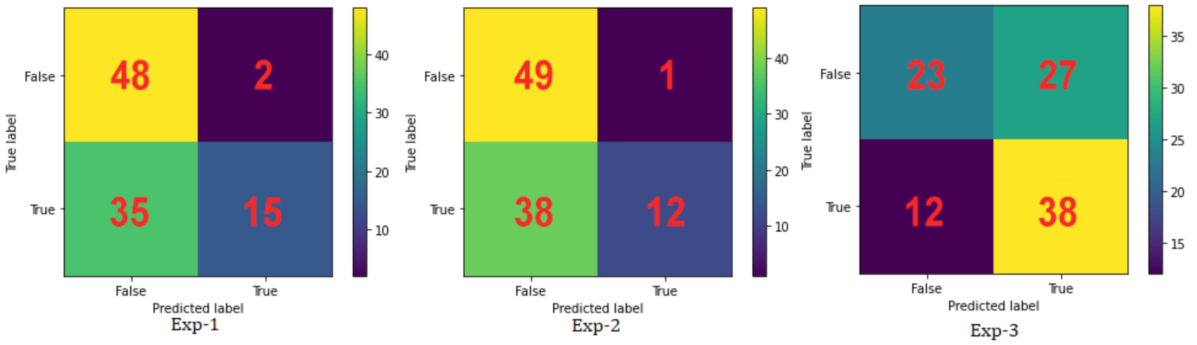

Figure 8. Model B Confusion Matrices using Fake Image Dataset

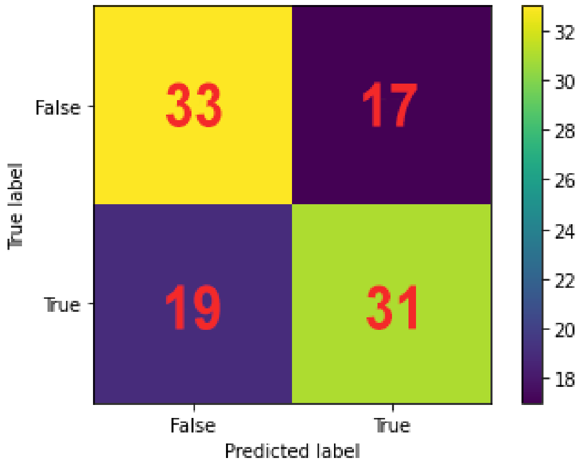

Figure 9. Model C Confusion Matrices using Fake image Dataset

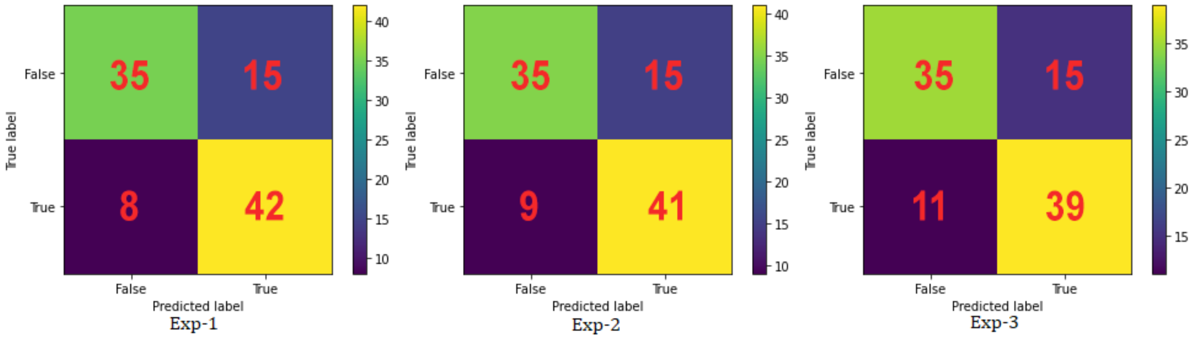

Figure 10. Model D Confusion Matrices using Fake Image Dataset

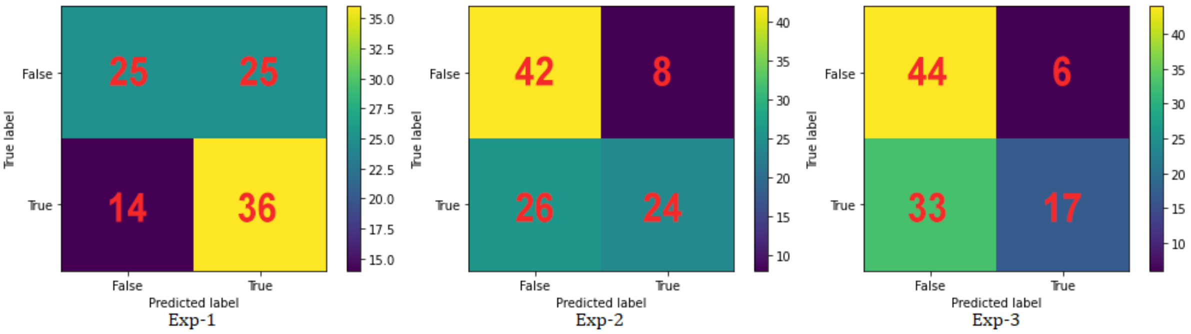

Figure 11. Model E Confusion Matrices using Fake Image Dataset

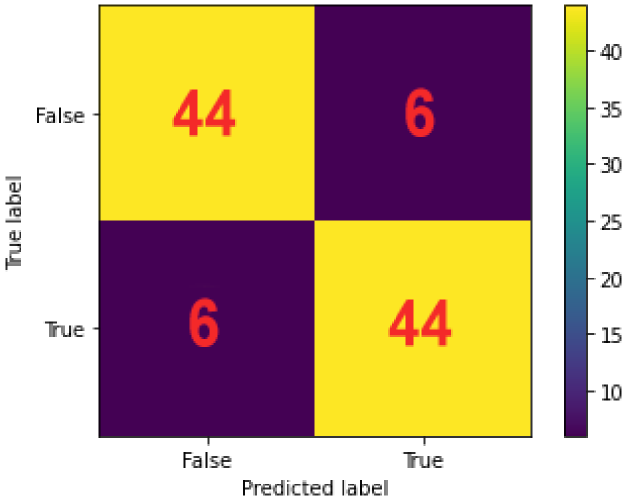

Figure 12. Model DR Confusion Matrix using Fake Image Dataset
